# Resveratrol attenuates the priapism phenotype in sickle cell mice by restoring NO-cGMP-PDE5 signaling and reducing NADPH oxidase 2 expression

**DOI:** 10.3389/fphar.2025.1551533

**Published:** 2025-04-30

**Authors:** Carolina Oliveira Splendore, Tammyris Helena Rebecchi Silveira, Dalila Andrade Pereira, Beatriz Pereira Bossarino, Mariana Gonçalves de Oliveira, Fabiano Beraldi Calmasini, Arthur L. Burnett, Fernando Ferreira Costa, Fábio Henrique Silva

**Affiliations:** ^1^ Laboratory of Pharmacology, São Francisco University Medical School, São Paulo, Brazil; ^2^ Universidade Federal de São Paulo, Escola Paulista de Medicina, Department of Pharmacology, São Paulo, Brazil; ^3^ The James Buchanan Brady Urological Institute and Department of Urology, The Johns Hopkins School of Medicine, Baltimore, MD, United States; ^4^ Hematology and Hemotherapy Center, University of Campinas, São Paulo, Brazil

**Keywords:** 3-nitrotyrosine, cGMP, hemoglobin, reactive oxygen species, corpus cavernosum

## Abstract

The pathogenesis of priapism in sickle cell disease (SCD) is closely linked to oxidative stress and reduced bioavailability of nitric oxide (NO) in penile tissue. Resveratrol, a potent natural antioxidant, has demonstrated protective effects in various vascular disorders. To evaluate the therapeutic effects of resveratrol on priapism, oxidative stress markers, and NO-cGMP signaling in the penile tissue of transgenic SCD mice. Male wild-type (C57BL/6) and transgenic SCD mice were treated with resveratrol (100 mg/kg/day, gavage) or vehicle for 2 weeks. Functional studies were conducted on CC strips mounted in organ baths to assess relaxation responses to acetylcholine (ACh), sodium nitroprusside (SNP), and nitrergic stimulation (electrical field stimulation, EFS). The oxidative stress markers (NOX-2, 4-HNE, and 3-NT), cGMP levels, and the mRNA expression of endothelial nitric oxide synthase (eNOS) and phosphodiesterase type 5 (PDE5) were evaluated. Resveratrol treatment decreased exaggerated ACh-, SNP-, and EFS-induced relaxation responses in SCD mice. It also reduced oxidative stress markers (NOX-2, 4-HNE, and 3-NT) and normalized eNOS and PDE5 mRNA expression in the CC of SCD mice. Additionally, cGMP levels in the CC were significantly increased by resveratrol treatment. These effects were specific to SCD mice and not observed in wild-type mice. In conclusion, resveratrol reduces oxidative stress and restores NO-cGMP signaling in the penile tissue, reducing the exaggerated cavernosal relaxation characteristic of priapism in SCD. These findings highlight resveratrol as a promising therapeutic candidate for managing priapism in patients with SCD.

## 1 Introduction

Sickle cell disease (SCD) is the most common genetic disease in the world, with an estimated 515,000 babies born annually in Africa, predominantly in sub-Saharan Africa, with 50%–90% dying before adulthood (Nkya et al., 2024). The condition is characterized by the presence of mutant hemoglobin S (HbS), which, under low oxygen conditions, promotes the polymerization of HbS and consequent deformation of erythrocytes into a sheet-like shape in the capillary beds (Kato et al., 2018). This morphological inheritance compromises efficient oxygen transport and predisposes patients to recurrent episodes of hemolysis and vaso-occlusive events. These manifestations have resulted in a wide range of acute and chronic complications, among which priapism stands out for its significant impact on quality of life (Kavanagh et al., 2022).

Ischemic priapism is a medical condition characterized by a prolonged and often painful erection of the penis that typically lasts for more than 4 h, regardless of sexual stimulation (Bivalacqua et al., 2022). Research encompassing adult males with SCD indicates a priapism prevalence of 32.6%, with the majority (74%) experiencing stuttering episodes, generally resolving within 4 h (Idris et al., 2020). Prolonged and untreated priapism can cause irreversible damage to penile tissue, including necrosis and fibrosis, culminating in irreversible erectile dysfunction (Salonia et al., 2014). Despite being recognized as a prevalent complication of SCD, the therapeutic options remain suboptimal, underscoring a need for more effective treatment modalities. Current clinical strategies, such as the use of low-dose sildenafil, have shown promise in reducing the frequency of recurrent priapism episodes in men with SCD without affecting normal erectile function (Burnett et al., 2006b; Burnett et al., 2006a; Burnett et al., 2014).

The primary molecular defect contributing to priapism in SCD is the diminished bioavailability of nitric oxide (NO)/cyclic guanosine monophosphate (cGMP) in the penis (Musicki and Burnett, 2020; Pereira et al., 2024a). This condition is attributed to diminished endothelial nitric oxide synthase (eNOS) activity along with increased reactive oxygen species (ROS), which readily neutralize NO (Champion et al., 2005; Musicki et al., 2011; Musicki et al., 2014; Musicki et al., 2018; Musicki et al., 2020; Bivalacqua et al., 2013; Silva et al., 2016a). The heightened oxidative stress observed in SCD penile tissue is linked with an upregulation of NOX-2 NADPH oxidase expression and the uncoupling of eNOS (Musicki et al., 2011; Musicki et al., 2014; Bivalacqua et al., 2013; Silva et al., 2016b; Pereira et al., 2022). Impaired signaling downstream from NO in SCD is linked with a diminished phosphodiesterase type 5 (PDE5) regulatory function, stemming from the lack of the cGMP-dependent feedback control mechanism. This deficiency leads to unchecked accumulation of cGMP following neurostimulation within the penile tissue, ultimately resulting in priapism (Champion et al., 2005; Lagoda et al., 2013; Silva et al., 2016b).

Resveratrol (3,5,4′-trihydroxystilbene) is a natural phytoalexin with potent antioxidant properties, commonly found in foods such as peanuts, grapes, and berries (Baur et al., 2006). Numerous studies have demonstrated the therapeutic potential of resveratrol in protecting against a range of diseases, including diabetes, cancer, hypertension, and cardiovascular disease, through its anti-hypertensive, cardioprotective, and endothelial protective effects (Bonnefont-Rousselot, 2016). In models of erectile dysfunction, resveratrol improved erectile function by improving endothelial function associated with increased eNOS expression, increased cGMP levels, and reduced production of ROS in the corpus cavernosum (Fukuhara et al., 2011; Sener et al., 2018; Yazir et al., 2018; Yu et al., 2022).

Given that oxidative stress impairs NO bioavailability and is implicated in the pathogenesis of priapism, we hypothesized that resveratrol treatment may reverse the oxidative stress and the priapism phenotype from transgenic SCD mice. Therefore, we have undertaken functional and molecular evaluations in this study to evaluate the therapeutic potential of resveratrol on priapism and the associated oxidative stress markers in penile tissue of SCD mice.

## 2 Materials and methods

### 2.1 Animals and treatment

All experimental procedures were approved by the Ethics Committee for the Use of Experimental Animals of the University of Campinas (UNICAMP). Three-to five-month-old wild-type (WT, C57BL/6) and Townes transgenic SCD mice were used. The animals were obtained from Jackson Laboratories (Bar Harbor, ME, United States) and bred and characterized at the Multidisciplinary Center for the Investigation of Biological Science in Laboratory Animals at UNICAMP. The homozygous Townes SCD model is a knock-in strain in which the murine α-globin genes are replaced with human α-globin genes, and the β-globin genes are replaced with human Aγ and βS (sickle) globin genes (Wu et al., 2006).

Mice were treated with resveratrol (100 mg/kg/day) or vehicle (water), administered once daily by oral gavage for 2 weeks. The gavage volume was adjusted to 10 µL/gof body weight.

### 2.2 Hematological parameters

Whole blood was collected by intracardiac puncture from mice anesthetized with ketamine (100 mg/kg) and xylazine (10 mg/kg), using EDTA-coated vacutainer tubes (BD Biosciences, Franklin Lakes, NJ, United States). Complete blood counts were performed within 30–60 min of collection using the Sysmex XN-3000™ hematology analyzer (Sysmex, Kobe, Japan). Following tissue collection, animals were euthanized by an overdose of isoflurane (12%).

### 2.3 Functional studies in cavernosal strips and concentration-response curves

CC tissues were collected from mice anesthetized with an intraperitoneal injection of ketamine (100 mg/kg) and xylazine (10 mg/kg). All efforts were made to minimize animal suffering. After tissue collection, animals were euthanized by an overdose of isoflurane (12%). CC from mice were mounted in a 5-mL organ system containing Krebs-Henseleit solution at 37°C and continuously bubbled with a mixture of 95% O_2_ and 5% CO_2_ (pH 7.4). Changes in isometric force were recorded using a strip myograph for isometric force recording (Model 610M, Denmark) coupled with an acquisition system (PowerLab 8/30, LabChart 7, ADInstruments, Sydney-NSW, Australia). At the start of the experiments, the resting tension was set to 2.5 mN. The corpus cavernosum strips were given a 60-minute equilibration period, during which the bathing medium was changed every 15 min. Cumulative concentration-response curves were constructed for both the muscarinic agonist acetylcholine (ACh, 10^−9^ to 10^−5^ M) and the NO-donor compound sodium nitroprusside (SNP; 10^−9^ to 3 × 10^−4^ M) in tissue strips pre-contracted with phenylephrine (3 × 10^−6^ to 10^−5^ M). EC_50_ values are presented as the negative logarithm (pEC_50_), and calculated by a fitting concentration–response relationship to a sigmoidal model of the form log-concentrations vs. response using the GraphPad Software (GraphPad Software, San Diego, CA, United States) (Pereira et al., 2024b).

### 2.4 Electrical-field stimulation (EFS) in corpus cavernosum strips

EFS was applied to the cavernosal tissues positioned between two platinum electrodes, connected to a Grass S88 stimulator (Astro-Med Industrial Park, RI, United States). The EFS was performed at a voltage of 50 V, with a pulse width of 1 m and trains of stimuli that lasted 10 s at varying frequencies. The frequency-response relationships were investigated using supra-maximum voltage in all electrically stimulated preparations. In order to study the nitrergic cavernosal relaxations, tissues were pretreated with guanethidine (3 × 10^−5^ M; to deplete the catecholamine stores of adrenergic fibers) and atropine (10^-6^ M; to produce muscarinic receptor antagonism) prior to pre-contraction with phenylephrine (3 × 10^−6^ to 10^−5^ M). When a stable contraction level was attained, a series of EFS-induced relaxations were constructed (2–32 Hz). Data were calculated relative to the maximal changes from the contraction produced by phenylephrine in each tissue, which was taken as 100%.

### 2.5 Western blot analysis

Total penile homogenates (50 μg total protein) were run on 4%–20% Tris-HCl gels (Bio-Rad Laboratories, Hercules, CA, United States), transferred to a polyvinylidene fluoride membrane, and incubated overnight at 4°C with the following antibodies: monoclonal anti-NOX-2 (1:1,000; BD Transduction Laboratories, catalog number 611414, San Diego, CA, United States), monoclonal anti-3-NT (1:1,000, Abcam, catalog number ab7048, Cambridge, MA), polyclonal anti-4-HNE antibody (1:1,000, catalog number ab46545, Abcam). Quantified densitometry results were normalized to GAPDH.

### 2.6 Real time reverse transcription polymerase chain reaction (RT-PCR)

Total RNA was extracted with Trizol Reagent (Invitrogen Corp., Carlsbad, Ca, United States) from mouse corpus cavernosum samples. Three microgram RNA samples were incubated with 1U DNaseI (Invitrogen, Rockville, MD, United States) for 15 min at room temperature (RT) and ethylenediaminetetraacetic acid was added to a final concentration of 2 mM to stop the reaction. The DNaseI enzyme was subsequently inactivated by incubation at 65°C for 5 min. DNaseI-treated RNA samples were then reverse transcribed with Superscript III and RNaseOut (Invitrogen Corp., Carlsbad, Ca, United States) for 50 min at 50°C, and 15 min at 70°C. cDNA samples were quantified using a Nanodrop spectrophotometer (ND-1000; Nanodrop Technologies Inc., Wilmington, DE, United States). Primers were designed using the PrimerExpressTM program (Applied Biosystems, Foster City, CA, USA) (Table 1). The ideal concentration of use was determined for each pair of primer and the amplification efficiency was calculated according to the equation *E* ^*(−1/slope)*^, to confirm the accuracy and reproducibility of the reactions. Amplification specificity was verified by running a dissociation protocol. qRT-PCRs were performed in duplicate, using 6 μL SYBR Green Master Mix (Applied Biosystems), 10 ng cDNA and ideal quantities of each primer in a final volume of 12 μL. Samples were run in MicroAmp Optical 96-well plates (Applied Biosystems) in a 7,500 Fast Real Time PCR System (Applied Biosystems). Gene expression data were normalized using the geNorm method, based on the geometric mean of two validated reference genes (β-actin and GAPDH) (Vandesompele et al., 2002). Results are expressed as relative mRNA expression levels.

**TABLE 1:** Sequences and ideal concentrations for the primers used in qRT-PCR.

Gene	Primer sequence	Concentration
*NOS3* – F *NOS3*– R	5′-CCCAGGAGAGATCCACCTCA-3′ 5′-CAGACACCGTAGTGCAGAGGG-3′	150 nM
*PDE5A* – F *PDE5A* – R	5′-GGAAATGGTGGGACCTTCACT-3′ 5′-AAGAACAATACCACAGAATGCCA-3′	150 nM
*ACTB* – F *ACTB* – R	5′-ACTGCCGCATCCTCTTCCT-3′ 5′-GAACCGCTCGTTGCCAATA-3′	70 nM
*GAPDH* – F *GAPDH* – R	5′-TGCACCACCAACTGCTTA-3′ 5′-GGATGCAGGGATGATGTTC-3′	70 nM

F, foward; R, reverse; eNOS, endothelial nitric oxide synthase; PDE5, phosphodiesterase type 5.

### 2.7 Statistical analysis

The statistical analysis was performed using the GraphPad Prism program (GraphPad Software Inc., San Diego, CA, United States). The data was presented as the mean ± standard error of mean (S.E.M.) of N experiments. To compare the statistical significance of the results, a one-way analysis of variance (ANOVA) was used, and the Tukey method was applied as a post-test. A P value of less than 0.05 was considered statistically significant.

## 3 Results

### 3.1 Treatment with resveratrol did not change hematological parameters in mice

**FIGURE 1:**
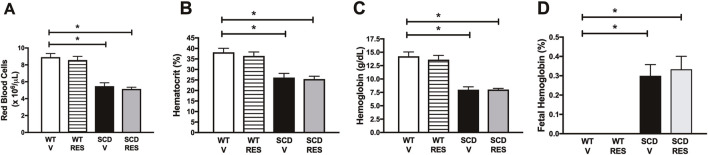
Hematological parameters in WT, SCD mice treated with vehicle (V) or resveratrol (RES). **(A)** Erythrocytes, **(B)** hematocrit, **(C)** total hemoglobin, **(D)** fetal hemoglobin. Data represent the means ± SEM of 10 animals. *P < 0.05 compared to control group.

The hematological parameters shown in Figure 1 demonstrate that mice with SCD have severe anemia, evidenced by a reduction in the number of red blood cells, hematocrit values, and hemoglobin levels when compared to the WT group. Additionally, fetal hemoglobin levels were significantly higher in the SCD group than in the WT group (Figure 1D). Treatment with resveratrol did not result in any changes in the evaluated hematological parameters (Figures 1A–D).

### 3.2 Resveratrol reduces increased ACh-induced cavernosal relaxation in SCD mice

The addition of phenylephrine (3 × 10^−6^ to 10^–5^ M) to the tissue bath caused submaximal contractions of cavernosal segments that did not significantly differ between groups (0.41 ± 0.02, 0.40 ± 0.03, 0.42 ± 0.03, and 0.43 ± 0.03 mN for WT + vehicle, WT + resveratrol, SCD + vehicle, and SCD + resveratrol groups, respectively; n = 15).

**FIGURE 2:**
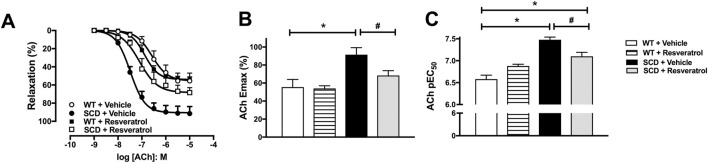
**(A)** Concentration-effect curves for acetylcholine (ACh) in corpus cavernosum of WT and SCD mice treated with resveratrol. **(B,C)** ACh maximum response (Emax) and potency (pEC_50_) values, respectively. Data represent the means ± SEM of 5 animals. *P < 0.05 compared to vehicle-control group; #P < 0.05 compared to SCD-Vehicle.

Endothelium-dependent relaxation was assessed by constructing concentration-effect curves for ACh (1 nM - 10 μM) in CC of mice pre-contracted with phenylephrine (3–10 μM) (Figure 2A). ACh potency (pEC_50_) and maximal response (E_max_) values were significantly higher (P < 0.05) in the CC of SCD mice compared to WT mice (Figures 2B,C). Treatment with resveratrol significantly (P < 0.05) reduced ACh potency and E_max_ in the CC of the SCD group (Figures 2B,C). However, neither the potency (Figure 2B) nor the Emax (Figure 2C) of ACh were altered in the CC of WT mice treated with resveratrol.

### 3.3 Resveratrol reduces increased SNP-induced cavernosal relaxation in SCD mice

**FIGURE 3:**
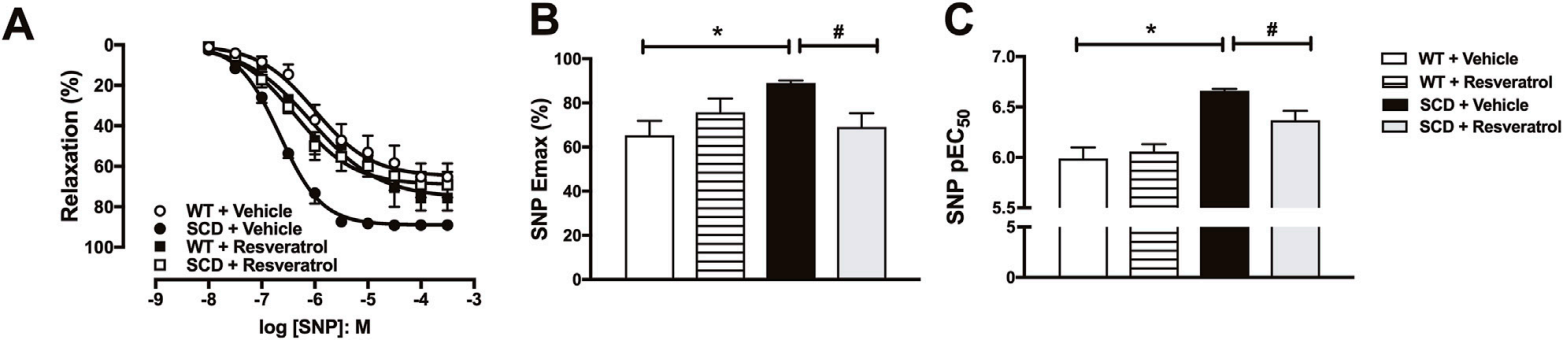
**(A)** Concentration-effect curves for sodium nitroprusside (SNP) in corpus cavernosum of WT and SCD mice treated with resveratrol. **(B,C)** SNP maximum response (E_max_) and potency (pEC_50_) values, respectively. Data represent the means ± SEM of 5 animals. *P < 0.05 compared to vehicle-control group; #P < 0.05 compared to SCD-Vehicle.

Endothelium-independent relaxation was assessed by constructing concentration-effect curves to SNP (10 nM −300 μM) in CC of mice pre-contracted with phenylephrine (3–10 μM) (Figure 3A). SNP pEC_50_ and E_max_ values were significantly higher (P < 0.05) in the CC of SCD mice compared to WT mice (Figures 3B,C). Treatment with resveratrol fully normalized (P < 0.05) the values of E_max_ (Figure 3B) and pEC_50_ (Figure 3C) of the SNP in CC of the SCD group to values similar to those of the WT. Neither the pEC_50_ (Figure 3B) nor the Emax of SNP (Figure 3C) were altered in the CC of WT mice treated with resveratrol.

### 3.4 Resveratrol reduces increased nitrergic relaxation-induced cavernosal relaxation in SCD mice

**FIGURE 4:**
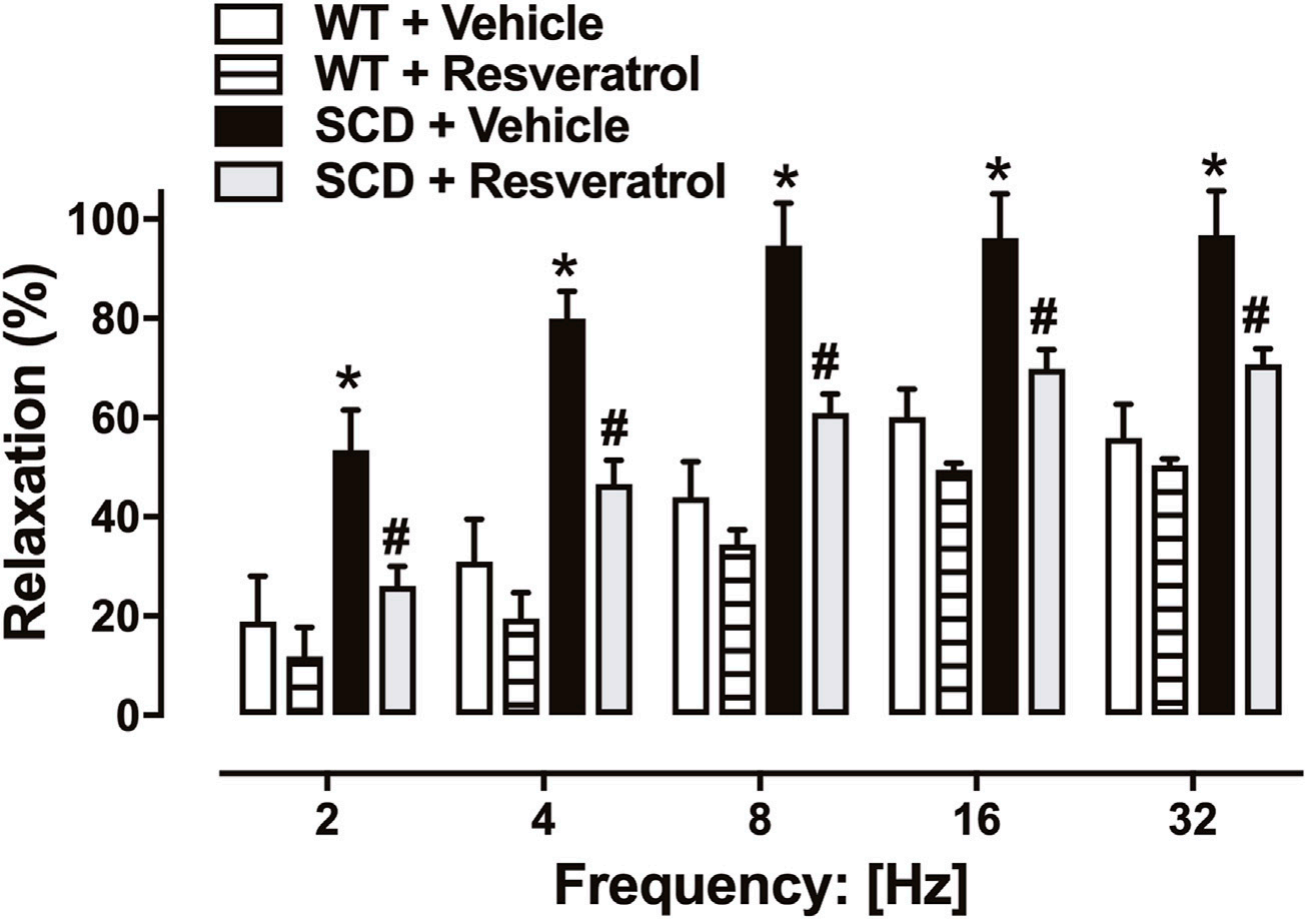
Relaxations of corpus cavernosum induced by EFS (2–32 Hz) in corpus cavernosum of WT and SCD mice treated with resveratrol or vehicle. Data represent the means ± SEM of 5 animals. *P < 0.05 compared to Control-Vehicle group; #P < 0.05 compared to SCD-Vehicle.

EFS induced frequency-dependent relaxations (2–32 Hz) in CC in all groups. Nitrergic relaxations were significantly greater in the CC of SCD mice at all studied frequencies (Figure 4). Treatment with the compound resveratrol normalized nitrergic relaxation in the SCD group but did not change it in the control group (Figure 4).

### 3.5 Resveratrol normalized the expression of eNOS and PDE5 mRNA in the corpus cavernosum of SCD mice

**FIGURE 5:**
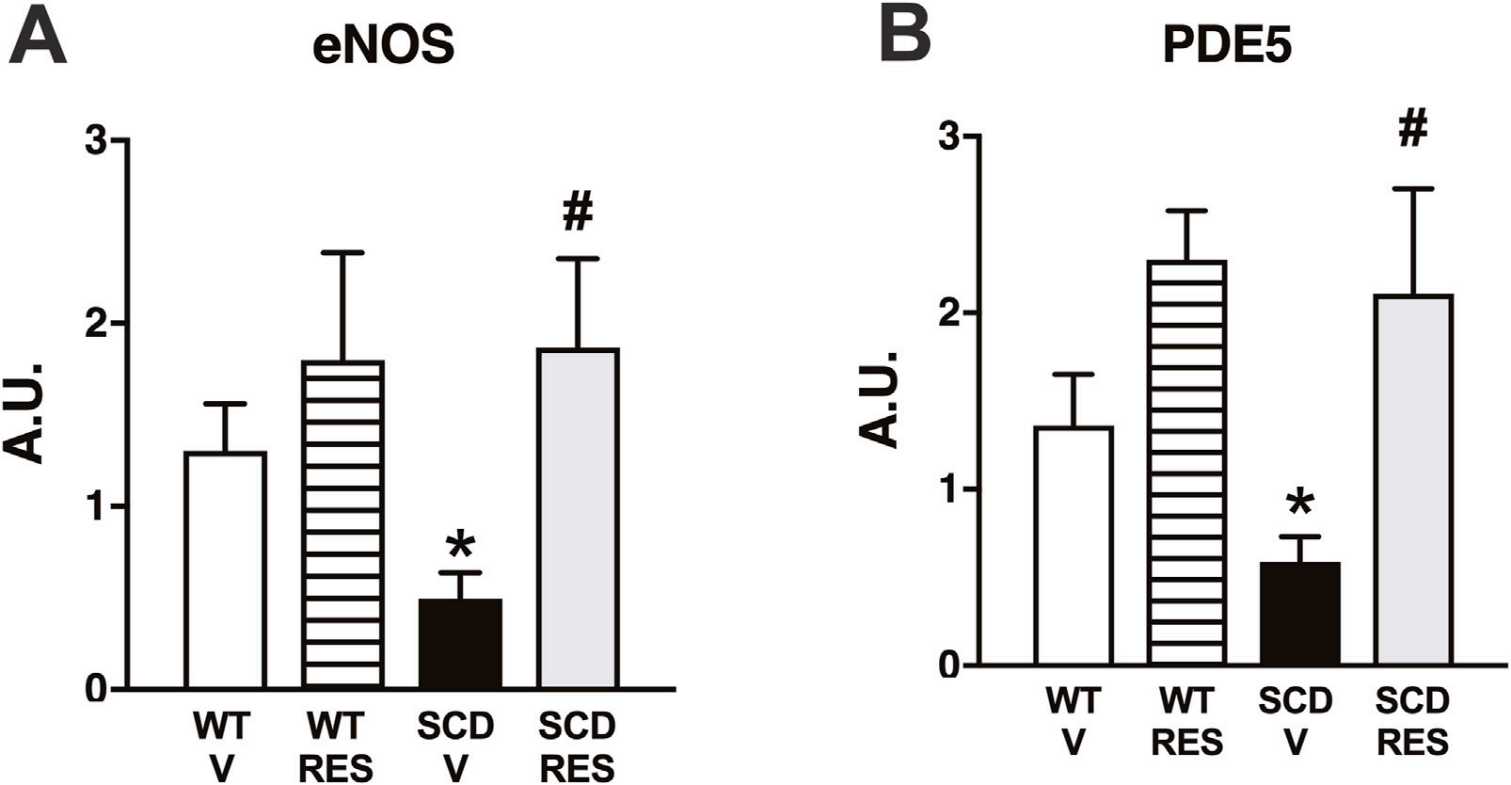
Expression of eNOS **(A)** and PDE5 **(B)** mRNA in corpus cavernosum of WT and SCD mice treated with resveratrol. Data are presented as mean ± SEM (n = 5–6) of arbitrary units (a.u.). *P < 0.05 compared with control group; #P < 0.05 compared to SCD-Vehicle. V, vehicle. RES, resveratrol.

Expression of eNOS and PDE5 mRNA was significantly lower in the SCD group compared to the WT group (Figures 5A,B). Treatment with resveratrol normalized eNOS and PDE5 mRNA expression in the CC of the SCD group, but did not change it in the WT group (Figures 5A,B).

### 3.6 Treatment with resveratrol normalized NOX-2 protein expression

**FIGURE 6:**
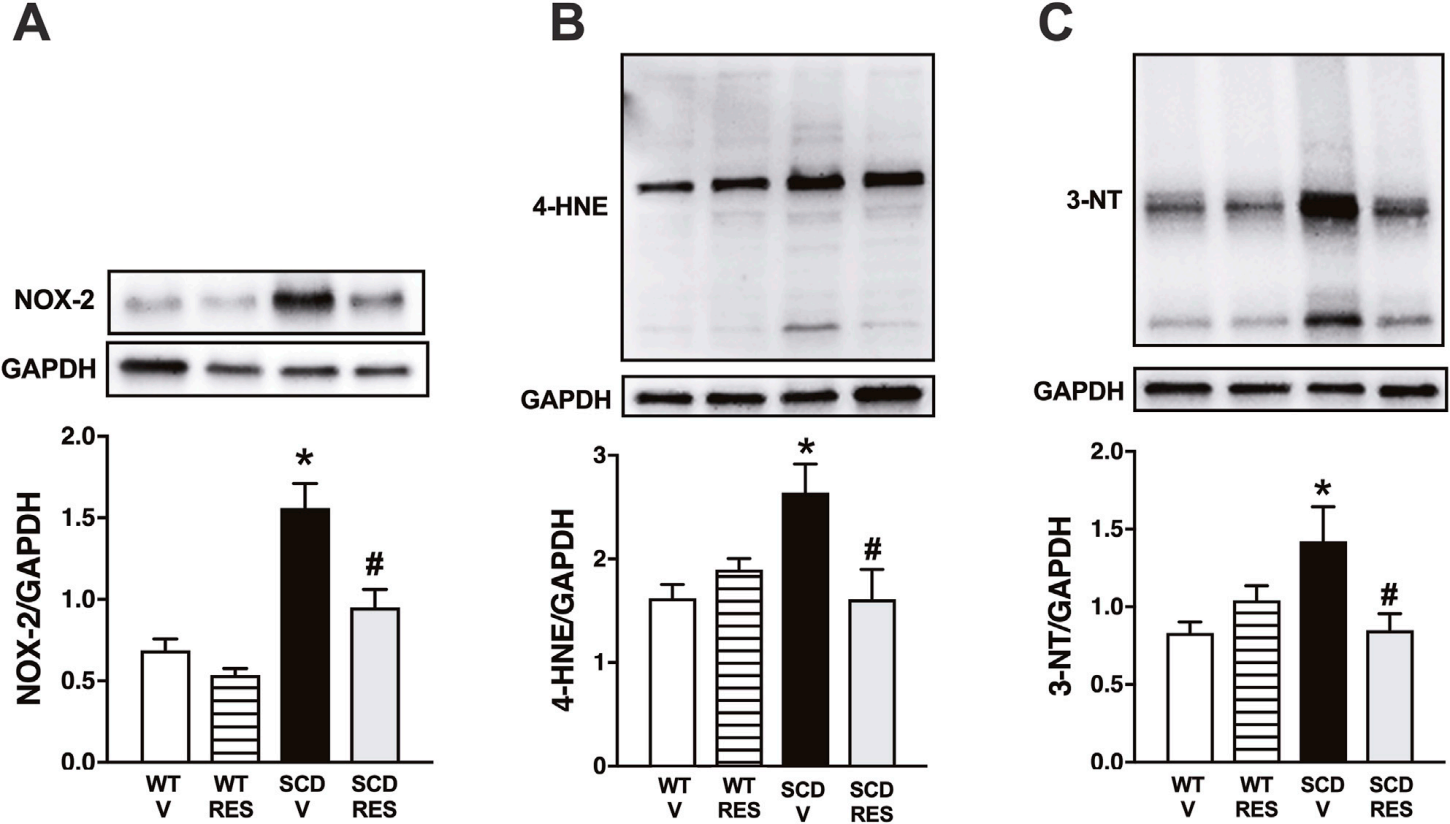
NOX-2 **(A)**, 4-HNE **(B)** and 3-NT **(C)** protein expression in CC from WT and SCD mice treated with resveratrol. Data represent the means ± SEM of 4-5 animals. *P < 0.05 compared with control group; #P < 0.05 compared to SCD-Vehicle.

NOX-2 protein expression was 129% higher (P < 0.05) in CC from the SCD group compared to the WT group (Figure 6A). Treatment with resveratrol normalized the NOX-2 protein expression in the CC of the SCD group, but did not modify it in the WT group.

### 3.7 Treatment with resveratrol normalized the protein expression of markers of oxidative stress and nitrosative stress in the corpus cavernosum of the sickle cell group

Protein expression of 4-HNE (Figure 6A) and 3-NT (Figure 6B) was 62% and 73% higher (p < 0.05) in the CC of the SCD group compared to the control group, respectively. Treatment with resveratrol normalized the protein expression of 3-NT and 4-HNE in the CC of the SCD group, but did not modify it in the WT group (Figures 6A,B).

### 3.8 Treatment with resveratrol increased cGMP levels in the corpus cavernosum of the sickle cell group

**FIGURE 7:**
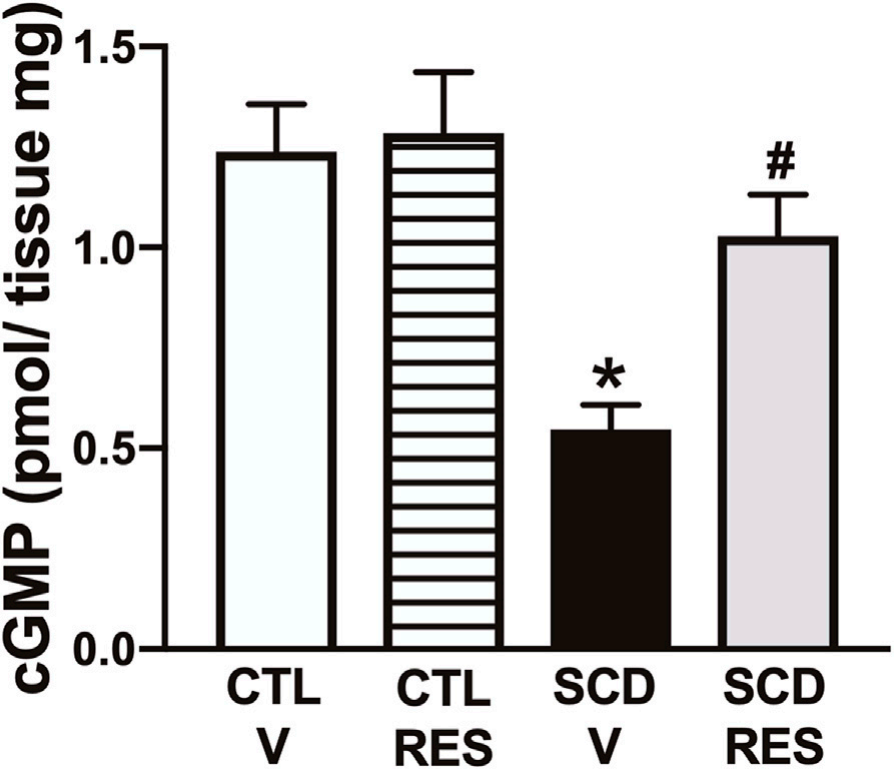
Basal GMP contents of penis from WT and SCD mice treated with resveratrol. Data represent the mean ± S.E.M. for 6 mice in each group. P < 0.05 compared with control group; #P < 0.05 compared to SCD-Vehicle. V, vehicle. RES, resveratrol.

The basal cGMP content in the erectile tissue was 56.4% lower (P < 0.05) in penises of SCD mice compared with WT-vehicle mice (Figure 7). Resveratrol treatment increased (P < 0.05) the cGMP levels in the penis of the SCD group, but did not modify it in the control group (Figure 7).

## 4 Discussion

The present study demonstrates that resveratrol exerts significant therapeutic effects on the penile tissue of SCD mice, primarily by modulating key pathways associated with oxidative stress and NO-cGMP signaling. Despite the severe anemia and altered hematological parameters characteristic of SCD, resveratrol treatment did not influence these systemic markers. Notably, resveratrol effectively reduced the exaggerated cavernosal relaxation responses to endothelium-dependent (ACh), endothelium-independent (SNP), and nitrergic (EFS) stimuli in SCD mice, indicating a restoration of normal penile smooth muscle function. This normalization was further supported by the upregulation of eNOS and PDE5 mRNA expression and the reduction of oxidative stress markers (NOX-2, 4-HNE, and 3-NT) in the corpus cavernosum. Additionally, the observed increase in cGMP levels in the erectile tissue of resveratrol-treated SCD mice underscores its role in maintaining the balance required for normal erectile function.

Penile erection is initiated by the relaxation of the corpus cavernosum smooth muscle (Burnett, 2006). The NO-cGMP signaling pathway plays a crucial role in this process, acting as the main inducer of erectile tissue relaxation (Andersson, 2011). The production of NO, which occurs in the endothelial cells and nitrergic fibers in the penis, is catalyzed by the enzymes eNOS and nNOS (Burnett, 2006). Upon production, NO activates sGC in the smooth muscle, converting GTP to cGMP and leading to the stimulation of cGMP-dependent protein (PKG), culminating in the relaxation of CC smooth muscle cells (Andersson, 2011). The duration of the erectile response is determined by the quick conversion of cGMP to 5′GMP by the enzyme PDE5 (Lin et al., 2002). In additional, cGMP has a crucial role in the regulation of PDE5 gene expression in the smooth muscle of the CC (Lin et al., 2002). Notably, the penile tissue of mice with SCD exhibits a reduction in PDE5 expression, which is attributed to the diminished basal bioavailability of NO-cGMP due to decreased eNOS expression and increased oxidative stress (Bivalacqua et al., 2013; Silva et al., 2016b; Musicki et al., 2018; 2020; Pereira et al., 2022). Consistent with previous research, our findings demonstrate that the priapism phenotype in SCD mice is characterized by enhanced smooth muscle relaxation of the CC in response to NO-cGMP pathway stimulants such as ACh, SNP, and EFS, attributed to reduced cGMP degradation by PDE5, resulting in prolonged and exaggerated penile erections (Silva et al., 2016b; Musicki et al., 2020; Pereira et al., 2022; Pinheiro et al., 2022).

An effective treatment strategy for priapism involves addressing the underlying pathophysiological mechanisms, particularly by enhancing the expression of eNOS. Numerous studies have shown that resveratrol can increase eNOS expression in both blood vessels and the corpora cavernosa, positioning it as a promising therapeutic candidate (Das et al., 2005; Arunachalam et al., 2010; Pektaş et al., 2015; Yazir et al., 2018; Feng et al., 2019; Li et al., 2019; Tasatargil et al., 2019). In our study, SCD mice exhibited reduced eNOS expression in the corpus cavernosum, an alteration that was effectively reversed by resveratrol treatment. This normalization of eNOS expression was accompanied by an upregulation of PDE5. Based on these findings, we propose that the restoration of eNOS function may directly influence PDE5 levels. Consequently, resveratrol treatment led to a significant reduction in the exaggerated cavernosal relaxation induced by NO-cGMP pathway stimuli (ACh, SNP, and EFS), which is consistent with the increase in PDE5 expression. These findings underscore the potential of resveratrol to correct the molecular imbalances contributing to priapism, offering a targeted approach to managing this condition.

SCD is associated with oxidative stress, which is characterized by an imbalance between the production and elimination of ROS (Sies et al., 2024). This results in oxidative stress in the penis of SCD patients (Lagoda et al., 2013) and mice (Musicki et al., 2012; Musicki et al., 2020; Silva et al., 2016b; Pereira et al., 2022). NOX-2 (aka gp91phox), a well-known NADPH oxidase isoform, is expressed in various cell types, including endothelial cells, and is a major source of superoxide anion in vascular tissues (Frey et al., 2009). In SCD mice, the upregulation of NOX-2 plays a crucial role in the pathophysiology of priapism (Lagoda et al., 2013; Lagoda et al., 2014; Musicki et al., 2014; Silva et al., 2016b; Pereira et al., 2022). This is because the excess of superoxide anion, which are produced by an upregulation of NOX-2, can lead to oxidative stress and subsequent lipid peroxidation. Lipid peroxidation is the process by which polyunsaturated fatty acids in cellular membranes are attacked by free radicals, causing cell membrane damage and the production of reactive aldehydes such as 4-HNE (Yang et al., 2003). 4-HNE is a highly reactive aldehyde that can modify proteins and other cellular components, leading to cellular damage and dysfunction (Yang et al., 2003). 4-HNE is widely used as an indicator of oxidative stress and its elevated levels are associated with priapism in SCD (Musicki et al., 2014; Pereira et al., 2022). Additionally, excessive superoxide anion concentration can react with NO, generating peroxynitrite, which is a powerful oxidant and cytotoxic agent that can lead to increased production of 3-nitrotyrosine (Radi, 2013). 3-nitrotyrosine is a stable marker of protein modification by peroxynitrite and is used as an indicator of oxidative stress and its elevated levels of 3-NT are associated with priapism in SCD (Lagoda et al., 2014; Silva et al., 2016b; Pereira et al., 2022). Resveratrol has been demonstrated to improve vascular and erectile function by reducing oxidative stress, which is achieved through the downregulation of the expression or activity of NOX-2 (Murat et al., 2016; Pierre et al., 2022), reduction of 4-HNE (Kaneko et al., 2011; Mattison et al., 2014) and 3-NT. Our study demonstrated that resveratrol treatment effectively reduced the elevated NOX-2 protein expression in the penile tissues of SCD mice. Decreased 4-HNE and 3-NT protein expression by resveratrol treatment is likely associated with lower superoxide anion production by NADPH oxidase in smooth muscle cavernosal cells of SCD. This reduction in oxidative stress likely resulted from lower superoxide anion production by NADPH oxidase, thereby improving the bioavailability of NO-cGMP and contributing to the normalization of PDE5 expression in the penile tissue of SCD mice.

Hydroxyurea was the first drug approved for the treatment of SCD, which increases the production of fetal hemoglobin within erythrocytes, thereby reducing the formation of HbS polymers and improving patient outcomes (Steinberg et al., 2003). Fetal hemoglobin is composed of two gamma chains and two alpha chains (α2γ2), and previous studies have shown that resveratrol is an inducer of gamma-globin chains, leading to increased HbF synthesis in cultured erythroid progenitor cells from SCD patients (Fibach et al., 2012). However, in our study, we found that treatment with resveratrol did not change HbF expression, number of red blood cells and hematocrit of SCD mice. These findings suggest that the beneficial effects of resveratrol on the penile tissue of SCD mice are not related to improvements in hematological parameters.

One limitation of our study is that we did not evaluate the effects of resveratrol on endogenous antioxidant defense systems, such as superoxide dismutase, catalase, or glutathione peroxidase (Truong et al., 2018). Resveratrol is known to modulate these enzymatic pathways, which may also contribute to the observed reduction in oxidative stress. Therefore, we cannot exclude the possibility that the antioxidant effects of resveratrol in SCD penile tissue are partially mediated by the upregulation of intrinsic antioxidant responses, in addition to the suppression of NOX-2 expression. Furthermore, plasma and penile tissue concentrations of resveratrol were not assessed in the present study. Future investigations should include pharmacokinetic analyses to determine the systemic and local concentrations achieved under the experimental conditions, and to better correlate exposure with the observed biological effects. Additionally, we did not investigate the acute effects of resveratrol in isolated tissue preparations. Studies evaluating the impact of resveratrol applied directly to the bath at physiologically relevant concentrations could help elucidate potential rapid, non-genomic effects on cavernosal reactivity and further clarify its pharmacological profile.

## 5 Conclusion

In summary, our study showed that resveratrol treatment reduces excessive cavernosal relaxation in SCD mice induced by the stimulation of the NO-cGMP pathway due to the normalization of PDE5 expression. Additionally, resveratrol treatment increased the cGMP bioavailability due to the downregulation of markers of oxidative stress, such as NOX-2, 3-NT, and 4-HNE, as well as the increased expression of eNOS in the penis of the SND group. Furthermore, the benefits of resveratrol appear to be localized to the erectile tissue, as the treatment did not alter systemic hematological parameters, including HbF levels. Overall, our results suggest that resveratrol may have therapeutic potential to modulate the priapism phenotype in patients with SCD, although further *in vivo* studies are required to confirm this effect.

## Data Availability

The original contributions presented in the study are included in the article/supplementary material, further inquiries can be directed to the corresponding author.
